# 4178 The feasibility/acceptability of using smartphone technology to assess mental health symptoms among Spanish-speaking outpatients

**DOI:** 10.1017/cts.2020.196

**Published:** 2020-07-29

**Authors:** Caroline Silva, Nilsa Ricci, Alessandra Pérez Mingoia, Taylor Myers, Kimberly Van Orden, Yeates Conwell

**Affiliations:** 1 University of Rochester Medical Center; 2 University of Rochester School of Medicine; 3 Carlos Albizu University - San Juan Campus

## Abstract

OBJECTIVES/GOALS: Geographic and linguistic isolation is associated with negative mental health outcomes, including increased risk for suicide, among ethnic/racial minorities. This study explores the feasibility of using smartphone technology with active and passive sensing to assess mental health symptoms and social behavior among at risk Spanish-speakers. METHODS/STUDY POPULATION: Participants were 13 Spanish-speaking adult outpatients who reported hopelessness/suicide ideation in the last month. Participants completed a baseline interview, 2-weeks of remote ecological momentary assessments (EMA; 4xday) using a smartphone with optional passive sensing (GPS, ambient sound recording), and a final interview. All participants identified as Hispanic (84.6% female, M age = 42.24 years). 53.8% identified as White, with 46.2% reporting race as Other (e.g., Indio, Trigueña). On average, participants had lived in the USA for 6.56 years (*SD* = 12.63 years). A majority (69.2%) met for Major Depressive Episode Current. At baseline, 53.8% reported passive and 23.1% reported active suicide ideation in the last month. 46.2% of participants reported a previous suicide attempt. RESULTS/ANTICIPATED RESULTS: A majority (84.6%) of participants consented to all passive data collection (GPS tracking and ambient sound recording). One participant dropped out after baseline and did not complete the EMA study portion. Participants completed on average 76.93% EMA survey instances (*SD* = 18.01%). Baseline depression/anxiety severity were significantly positively associated with symptom severity at 2-week follow-up (*p* < .01); however, baseline suicide ideation was not associated with ideation at follow-up. Participants did not report significant changes in mood or ideation from baseline to 2-week follow-up. Symptom severity at baseline was not associated with EMA instances completed. Percent of EMA instances completed were also not associated with symptom severity at follow-up, controlling for baseline severity. DISCUSSION/SIGNIFICANCE OF IMPACT: Results support the feasibility of smartphones to assess mental health symptoms and behaviors in real time, real world settings with Spanish-speakers. A majority of patients consented to active and passive remote assessments. Adherence to remote EMA was good and study attrition was minimal. Implications and future directions will be discussed.

